# Volitional control of the anterior insula in criminal psychopaths using real-time fMRI neurofeedback: a pilot study

**DOI:** 10.3389/fnbeh.2014.00344

**Published:** 2014-10-14

**Authors:** Ranganatha Sitaram, Andrea Caria, Ralf Veit, Tilman Gaber, Sergio Ruiz, Niels Birbaumer

**Affiliations:** ^1^Institute of Medical Psychology and Behavioral Neurobiology, University of TübingenTübingen, Germany; ^2^Department of Biomedical Engineering, University of FloridaGainesville, FL, USA; ^3^Sri Chitra Tirunal Institute of Medical Sciences and TechnologyThiruvananthapuram, Kerala, India; ^4^Departamento de Psiquiatría, Escuela de Medicina, Centro Interdisciplinario de Neurociencias, Pontificia Universidad Católica de ChileSantiago, Chile; ^5^Ospedale San Camillo, Istituto di Ricovero e Cura a Carattere Scientifico (IRCCS)Venezia, Italy

**Keywords:** real-time fMRI, brain-computer interface, neurofeedback, criminal psychopathy, antisocial behavior, anterior insula

## Abstract

This pilot study aimed to explore whether criminal psychopaths can learn volitional regulation of the left anterior insula with real-time fMRI neurofeedback. Our previous studies with healthy volunteers showed that learned control of the blood oxygenation-level dependent (BOLD) signal was specific to the target region, and not a result of general arousal and global unspecific brain activation, and also that successful regulation modulates emotional responses, specifically to aversive picture stimuli but not neutral stimuli. In this pilot study, four criminal psychopaths were trained to regulate the anterior insula by employing negative emotional imageries taken from previous episodes in their lives, in conjunction with contingent feedback. Only one out of the four participants learned to increase the percent differential BOLD in the up-regulation condition across training runs. Subjects with higher Psychopathic Checklist-Revised (PCL:SV) scores were less able to increase the BOLD signal in the anterior insula than their lower PCL:SV counterparts. We investigated functional connectivity changes in the emotional network due to learned regulation of the successful participant, by employing multivariate Granger Causality Modeling (GCM). Learning to up-regulate the left anterior insula not only increased the number of connections (causal density) in the emotional network in the single successful participant but also increased the difference between the number of outgoing and incoming connections (causal flow) of the left insula. This pilot study shows modest potential for training psychopathic individuals to learn to control brain activity in the anterior insula.

## Introduction

Psychopathy is a personality disorder described by a constellation of affective, interpersonal and behavioral characteristics such as callousness, a marked lack of empathy, egocentricity and impulsivity. Psychopaths engage in more criminal behavior and institutional misconduct than their non-psychopathic counterparts. Central to psychopathy is the deficient processing of emotions. These include shallowness and profound lack of remorse or empathy. Hare et al. (1991) subsumed those features under the term “emotional detachment”. Lykken (1957), using questionnaires and electrodermal responses, investigated the hypothesis that psychopaths fail to develop anxiety. Lykken found reduced anxiety levels in the subjective evaluations and low electrodermal responses to conditioned stimuli that were previously associated with shock in the autonomic indices. Cleckley (1951) suggested that psychopaths exhibit discordance in the verbal and experiential components of emotion. Empirical evidence indicates that psychopathic individuals have less intense aversive emotional reactions to many everyday situations than do non-psychopaths (Day and Wong, 1996). Other investigators suggested that the inability of psychopaths to anticipate the negative consequences of their behavior results from an insufficient capacity to develop anticipatory fear (Hare, 1978). Hence, psychopathy may be characterized by a faulty modulation of associative links between external stimuli and internal reactions (Patrick, 1994; Patrick et al., 1994). Neuroimaging studies investigating the affective processing of psychopathy will potentially lead to an understanding of neural mechanisms and elements that maintain this disorder (Porter, 1996). We have reported that the absence of conditioned fear in psychopathic individuals is reflected in a virtually complete lack of activation of the fear circuitry in the brain (i.e., insula, anterior cingulate, amygdala, orbital frontal cortex) (Birbaumer et al., 2005).

The amygdala plays a central role in emotional processing, particularly in fear conditioning (Kim and Jung, 2006). However, fear retention is not necessarily at the same site as fear association, and hence it is unclear whether the amygdala is the permanent storage site for long-term fear memory. Fear retention is abolished after amygdala lesion 1 day after, but not after many days, of inhibitory avoidance training, suggesting that long-term fear memory is not stored in amygdala (Liang et al., 1982; McGaugh et al., 1982). Electrical stimulation of the amygdala can have positive and negative emotional effects (Aggleton, 2000), and amygdala is active in conditions involving both positive and negative stimuli. In addition to the amygdala, a network of structures that includes insula, anterior cingulate gyrus and medial orbitofrontal cortex is suggested as important in identifying the emotional significance of the stimulus, and regulate the affective state (Adolphs, 2003a,b; Phillips et al., 2003). The insula has afferent and efferent connections to the medial and orbitofrontal cortices, anterior cingulate and amygdala (Augustine, 1996). Stein et al. (2007) maintain that the insula may have been relatively neglected compared with amygdala in the literature.

Insula activation is involved in several types of emotional processes, including differential positive vs. negative emotional processing (Büchel et al., 1998; Morris et al., 1998a,b), pain perception (Gelnar et al., 1999; Peyron et al., 2000), anticipation and viewing of aversive images (Simmons et al., 2004; Phan et al., 2006), and the making of judgments about emotions (Gorno-Tempini et al., 2001). Blair et al. (2006) propose that psychopathic individuals receive markedly reduced augmentation of the representation of the conditioned stimulus (CS) from the reciprocal connections of the amygdala and insula. Response to the CS will be impaired in individuals with psychopathy relative to controls (i.e., a weaker representation should be less able to control behavior). On the other hand, if the CS functions only as a distracter to ongoing behavior, performance will be superior in individuals with psychopathy relative to comparison individuals (i.e., a weaker representation will be a less of a competitor for the stimulus that should be controling behavior). Furthermore, insula is instrumental in the detection and interpretation of certain internal bodily states (Critchley et al., 2002, 2004), and is part of an interconnected subcortical network involved in empathic behavior (Decety et al., 2013).

Recent studies have provided new information on insula’s role in criminal and psychopathic behavior. de Oliveira-Souza et al. (2008) observed bilateral gray matter reductions in mid-anterior insula of community patients high on psychopathy scores compared with healthy individuals. Tiihonen et al. (2008) compared violent offenders with healthy subjects showing that the first group, and specially those with a diagnosis of psychopathy, had reductions of focal gray matter volumes in right insula. Similarly, Cope et al. (2012) examined a large sample of individuals from community correction centers observing that psychopathic traits were negatively associated with gray matter volumes in right insula. Schiffer et al. (2011) observed that violent offenders displayed smaller gray matter volume in the left insula, compared with non-offenders. Congruently with previous reports, left insula was the area of greatest difference between psychopath and non-psychopath prison inmates (psychopaths displayed significant insula cortical thinning) in a recent study by Ly et al. (2012). These findings are in line with a series of functional MRI studies that have shown hypoactivity of insula cortex on psychopathic individuals during classical fear conditioning as compared with healthy subjects (Veit et al., 2002; Birbaumer et al., 2005), that could act as a marker of psychopathy.

Until present it is unclear whether the defective brain fear circuitry of psychopaths can be modified at all. The stability of the psychopathic personality trait and its strong genetic determination (Tielbeek et al., 2012; Yildirim and Derksen, 2013) would argue against the possibility to self-regulate brain activity in fear-related regions. On the other hand, social and developmental factors also play a fundamental role in psychopathic behavior (Viding, 2004; Moffitt, 2005). These environmental factors suggest that modification of the fear circuitry is possible. In view of the above line of argument, we asked the following questions: could psychopaths be trained to regulate the BOLD activity in the insula, and does volitional increase of BOLD in insula have any effect on emotional processing? In this pilot study, we also wanted to test whether training to regulate the insular cortex changes the functional connectivity of the emotional network. We hypothesized that with learned increase of activity in the insula, connectivity to the other areas of the fear circuitry will improve.

We implemented a Functional Magnetic Resonance Imaging based Brain-Computer Interface (fMRI-BCI) for this study. An fMRI-BCI is a non-invasive system that can be used for on-line neurofeedback of the BOLD signal to learn regulation of localized changes in brain activity, and to study related behavioral effects of regulation of specific brain regions and their connectivity (Sitaram et al., 2007, 2009; Weiskopf et al., 2007; Caria et al., 2012; Birbaumer et al., 2013; Ruiz et al., 2013a, 2014; Sulzer et al., 2013). In a series of studies on healthy individuals, we investigated the self-regulation of anterior insula and its behavioral effects (Caria et al., 2007, 2010; Veit et al., 2012). The present pilot study employs a similar paradigm of feedback-guided regulation of left anterior insula and a subsequent evaluation of emotional and neutral pictures. Statistical parametric mapping and region of interest (ROI1) analyses were carried out to assess the brain activation during regulation, picture presentation and rating conditions. Statistical analysis was also carried out on the valence and arousal ratings of pictures to test for the influence of regulation of insular activity on picture processing.

For analyzing the functional connectivity of the emotional network due to regulation training, we used Granger Causality Modeling (GCM). Granger Causality Modeling is a method of vector autoregressive modeling using the theory of Granger causality to analyze directed influences (Goebel et al., 2003; Roebroeck et al., 2005; Seth, 2005, 2010; Abler et al., 2006). The method works by using temporal information in one or more time-series of brain regions to predict signal time courses in another region. These predictions can be used to arrive at temporally directed influences rather than only correlations in activity between brain regions. Directed influences of one neural system on another, called effective connectivity (Büchel and Friston, 2000; Friston and Büchel, 2000), could be generated for one or more regions of interest to develop a circuit diagram that could replicate the observed timing relationships between brain regions. We tested whether different stages of self-regulation training would indicate a change in the functional connectivity of the network.

## Methods

### Participants

Four psychopaths with criminal records participated in the study. Mean age of the psychopaths was 31.5 years (SD = 3.5 years, see Table 1). The criminal psychopaths were sexual offenders out on bail and waiting for their trial or out of jail and on parole, all of whom were screened from a larger sample using the Psychopathy Checklist: Screening Version (PCL:SV, Hart et al., 1995) and Levenson Self-Report Psychopathy Scale (LSRP; Levenson et al., 1995; Miller et al., 2008). The screening version of the PCL consists of 12 items and was developed to measure psychopathy in civic or forensic settings. Participants’ PCL:SV scores, including, emotional detachment aspects (PCL-SV F1) and antisocial behavior aspects (PCL-SV F2); and the LSRP scores, including, interpersonal and affective aspects (LSRP F1), and the social deviance aspects (LSRP F2) are listed in the Table 1. These values are much lower than the scores for the American population of psychopaths but are in accordance with the lower values for the German norms (Ullrich et al., 2003). The PCL:SV and LSRP scores were obtained by structured interviews conducted by the certified clinical psychologist of this study (author Niels Birbaumer). Written instructions were provided to all participants and informed written consent was obtained. The ethics committee of the Faculty of Medicine of the University of Tübingen approved the study.

**Table 1:** **The table reports for each participant his/her name, age, psychopathy score based on Psychopathy Checklist: Screening Version (PCL:SV, Hart et al., 1995) for factors emotional detachment (F1) and antisocial behavioral aspect (F2), and Levenson Self-Report Psychopathy Scale (LSRP, Levenson et al., 1995) for interpersonal and affective factor (LSRP F1), the social deviance factor (LSRP F2) and the total LSRP score**.

Participant	Age	PCL:SV Total	PCL:SV F1	PCL:SV F2	LSRP F1	LSRP F2	LSRP Total
GM	35	21	11	10	46	17	63
WE	35	7	03	04	31	16	47
RS	28	14	06	08	41	28	69
AK	28	6	02	04	20	26	46
**Mean**	**31.5**	**12**	**5.5**	**6.5**	**34.5**	**21.75**	**56.25**

### fMRI data acquisition

Functional images were acquired on a 3.0 T whole body scanner, with standard 12-channel head coil (Siemens Magnetom Trio Tim, Siemens, Erlangen, Germany). A standard echo-planar imaging sequence was used (EPI; TR = 1.5 s, matrix size = 64 × 64, effective echo time TE = 30 ms, flip angle α = 70°, bandwidth = 1.954 kHz/pixel). Sixteen slices (voxel size = 3.3 × 3.3 × 5.0 mm^3^, slice gap = 1 mm), AC/PC aligned in axial orientation were acquired. For superposition of functional maps upon brain anatomy a high-resolution T1-weighted structural scan of the whole brain was collected from each subject (MPRAGE, matrix size = 256 × 256, 160 partitions, 1 mm^3^ isotropic voxels, TR = 2300 ms, TE = 3.93 ms, TI = 1100 ms, α = 8°). In order to reduce movement artefacts, two foam cushions were used to immobilize the participant’s head.

### Localization of region of interest

A localizer run was used to delineate the target ROI1, the left anterior insula. A block-based paradigm was used for the localizer run consisting of four blocks (22.5 s each) during which the subjects had to use mental imagery to recall emotionally relevant personal experiences alternating with five baseline blocks (22.5 s each) during which they had to count in a reverse order from number 100. No verbal cues for the direction of emotional valence of mental strategies and imagery were given. Region of Interest, consisting of a rectangular area encompassing 5 × 5 voxels (~20 × 20 mm) on a single slice (5 mm), was selected based on the activation maps generated on-line during the task by means of Turbo-BrainVoyager (Goebel, 2001) and saved for the following training runs. The reference ROI2 was a large background ROI1 selected from a slice positioned distant from ROI1 encompassing a complete slice, selected with the intent to cancel global effects and to average out any unspecific activations. The ROI2 was chosen closer to the motor cortical surface to subtract movement related activations and further away from the anterior insula and other subcortical emotional brain regions.

### Experimental protocol

#### Feedback training

Each participant was conducted through three different types of protocols in this chronological order: a pretest on the first day; four feedback runs per day for 1–3 days depending on the availability of the subject; and a final day of a post-test. Each feedback run consisted of seven baseline blocks and six up-regulation blocks, each block of 30 s duration, presented in an alternating manner. During the up-regulation subjects had to increase the BOLD response in the ROI1 (left anterior insula) and during the baseline block return it to original value by watching the feedback presented in the form of an animated graphical thermometer. Normalized average BOLD signal from the left anterior insula was used to generate animated images of the varying thermometer bars. The feedback signal was computed as (BOLDupreg − BOLDbaseline)_ROI1_- (BOLDupreg − BOLDbaseline)_ROI2_. Where BOLDupreg and BOLDbaseline constituted the BOLD signal during the current scan of up-regulation, and the average BOLD signal from the previous baseline block, respectively. The up-regulation and baseline blocks were cued with red and blue colored backgrounds, respectively. Each run of the feedback training took 6.75 min to complete. At the end of each training run monetary reward was computed and presented to the subject proportional to the aggregate valid differential BOLD increase or decrease at every time point with respect to the previous time points. For computing monetary reward, percent BOLD change was counted as valid only if there was a BOLD increase but not decrease during the up-regulation block, and a percent BOLD decrease but not increase during the baseline block. Each valid percent increase and decrease of BOLD earned 10 European cents. Maximum reward for a feedback run was limited to 10 Euros.

#### Pre-test and post-test

**Figure 1:**
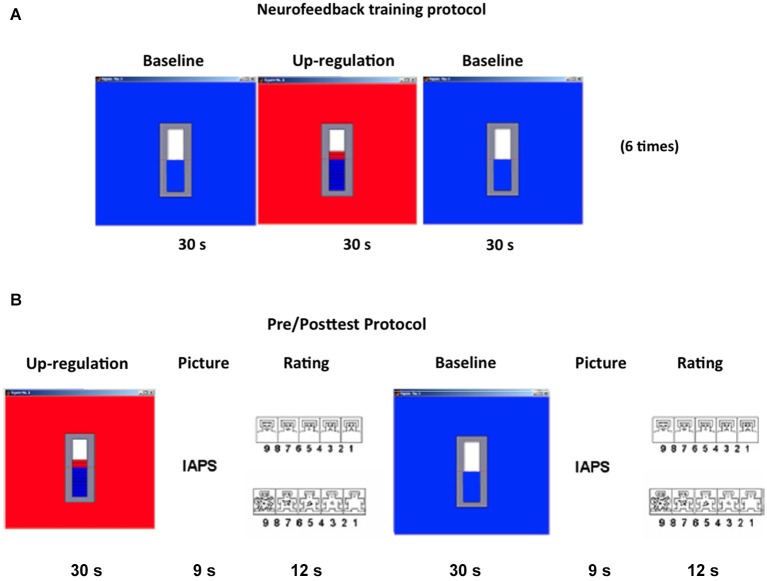
**Experimental design. (A)** RtfMRI feedback Training: Each feedback run consisted of seven baseline blocks and six up-regulation blocks, each block of 30 s duration, presented in an alternating manner. **(B)** Pre/Posttests: Each pre- or post-test consisted of five regulation blocks (30 s) alternating with six baseline blocks (30 s) both followed by an International Affective Picture System (31) picture presentation block (9 s) and a rating block (12 s). During rating blocks, participants were shown the Self-Assessment Manikin (34), which allows them to evaluate emotional valence and arousal. Both valence and arousal dimensions vary along a 9-point scale. Selection of the subjective rating was performed by positioning a red outline on the chosen number on each of the two scales, presented in close succession. Subjects were provided with two buttons allowing movements of the cursor in the left and right direction. (IAPS = International Affective Picture System).

The pre-test and post-test were similar in structure and only differed in the stimulus material used. The intent of the tests was to measure the effect of volitional regulation of the left anterior insula on aversive and neutral picture evaluation. Each run of the pre-test or post-test consisted of five alternating up-regulation and baseline runs, totally lasting 9 m. Each run consisted of a 30 s up-regulation or baseline block performed by watching the feedback of the moving thermometer bars as described before, followed by a 9 s emotional picture presentation block, that in turn was followed by a 12 s picture rating block (Figure 1A).

During picture presentation blocks, one emotional or neutral picture from the International Affective Picture System (IAPS; Lang et al., 2008, NIMH Center for the Study of Emotion and Attention, see Table 2) was presented. The pictures presented to the participants consisted of 20 aversive and 20 neutral pictures from IAPS. Valence and arousal ratings for aversive pictures were based on ratings from a large representative reference sample (Lang et al., 2008, NIMH Center for the Study of Emotion and Attention) with mean values of 3.26 ± 0.78 SD and 4.93 ± 0.47 SD, respectively. Valence and arousal ratings for neutral pictures had mean values of 4.84 ± 0.32 SD and 2.33 ± 0.40 SD, respectively. Pictures were pseudo-randomized such that no significant difference in standard ratings of valence and arousal was present between pictures after up-regulation and baseline blocks and between runs.

**Table 2:** **Valence and arousal ratings of emotion and neutral pictures shown during pre- and post-tests (Lang et al., 2008, NIMH Center for the Study of Emotion and Attention)**.

Pictorial stimuli from the International Affective Pictures System (IAPS)							
Aversive pictures				Neutral pictures			
IAPS number	Description	Valence	Arousal	IAPS number	Description	Valence	Arousal
9421	Soldier	2,47	4,86	5740	Plant	5,07	2,36
9430	Burial	3,1	4,81	7175	Lamp	4,78	1,55
9432	Mastectomy	3,29	4,12	7002	Towel	4,91	2,99
9433	Dead man	2,39	5	7950	Tissue	4,62	2,3
9440	Skulls	4,42	4,71	7020	Fan	5,02	2,15
9452	Gun	3,62	4,5	7025	Stool	4,46	2,44
9470	Ruins	3,62	4,53	7031	Shoes	4,2	1,67
9480	Skull	4,06	5,15	7010	Basket	4,95	1,55
9490	Corpse	4,2	5,41	7004	Spoon	4,89	2,09
9500	Porpoises	2,85	5,65	7224	File cabinets	4,38	2,55
9520	Kids	2,14	5,45	7217	Clothes rack	4,63	2,31
9530	Boys	3,65	4,74	7705	Cabinet	4,75	2,4
9560	Duck in oil	2,07	5,46	7080	Fork	5,43	1,98
9561	Sick kitty	3,2	4,18	7009	Mug	4,96	2,69
9570	Dog	1,9	5,84	7030	Iron	4,82	2,76
9571	Cat	2,65	4,68	7035	Mug	4,81	2,56
9582	Dental exam	4,09	5,15	7040	Dust pan	4,72	2,46
9584	Dental exam	3,44	4,65	7050	Hair dryer	4,81	2,59
9592	Injection	4	5,11	7140	Bus	5,59	2,67
9594	Injection	4,08	4,55	7233	Plate	5,01	2,51

During the evaluation blocks subjects had to rate the picture for valence and arousal using a button-based control device inside the MRI scanner. Pictures were rated in terms of subjective emotional valence and arousal using the Self-Assessment Manikin (SAM; Bradley and Lang, 1994). The SAM is a non-verbal pictorial assessment for measuring pleasure, aversion and arousal associated with a person’s affective reaction to a variety of stimuli. Valence and arousal dimensions vary along a 9-point scale. Before the experiment, participants were briefed about the experimental tasks, SAM ratings, and were also trained on how to rate the pictures using the two buttons. Positioning a red outline on the desired number of the SAM scale selected the appropriate subjective rating (Figure 1B).

### Off-line data analysis

#### SPM analysis of brain images

Off-line image post-processing and data analysis were performed using SPM2 statistical parametric mapping software package (Wellcome Department of Imaging Neuroscience, London), while BrainVoyager QX was used for ROI analysis. During signal preprocessing, the functional EPI volumes were realigned spatially, normalized into Montreal Neurological Institute (MNI) space, and smoothed spatially (9-mm Gaussian kernel) and temporally (0.0039 Hz, 1/(2.5 times the duration of the up-regulation and baseline block)) to remove high frequency artefacts. Hemodynamic response amplitudes were estimated using standard regressors, constructed by convolving a boxcar function, representing the block duration, with a canonical hemodynamic response function using standard SPM2 parameters. Motion parameters were also included into the general linear model (GLM) as covariates to take account for variance caused by head motion. Signal change during increase blocks with respect to the decrease blocks was evaluated by SPM2. Areas showing training related changes were analyzed with *t*-test comparisons of BOLD magnitude over runs.

#### Region of interest analysis

Hypothesis driven ROI analysis was performed using the ROI previously selected for each subject during the training. Region of Interest time series underwent the same preprocessing and model estimation using the GLM for whole brain analysis. The percent signal change during up-regulation blocks with respect to the baseline blocks was calculated for each run separately, and then also averaged across subjects. The training effect was evaluated by computing paired *t-*test on all subjects of percent signal changes in the target ROI run by run. Activation maps produced by offline analysis matched and validated activations maps produced in real-time by Turbo-BrainVoyager.

#### Analysis of picture ratings

Ratings of the IAPS pictures presented after increase blocks were compared with the ratings of the pictures presented after baseline blocks across runs. Local brain activity was also compared between pictures presented after increase blocks with respect to those presented after baseline blocks across runs.

#### Effective connectivity analysis

Granger Causality Modeling (Granger, 1969, 1980) is a method originally developed in economics for causal interaction between multiple events from time-series data. Recently, GCM has been applied in conjunction with Vector Autoregressive Models (VAR) to fMRI data also (Roebroeck et al., 2005; Abler et al., 2006) to investigate directed influences between neuronal populations. In this study, GCM analysis was carried out to evaluate the network dynamics during self-regulation during three different training runs: a run of weak regulation (2nd training run), a run of intermediate regulation (5th training run), and a run of strongest regulation (11th training run). Time-series of ROIs, known to be involved in self-induced or stimulus-induced emotions from literature (Phan et al., 2002) and also passed the height threshold of *P* < 0.05 (Bonferroni corrected) and a cluster threshold of 50 voxels were used as input to the GCM analysis for each stage of regulation. We implemented a multivariate GCM by adapting the Causal Connectivity Matlab (Mathworks Inc., USA) Toolkit from Seth (2005) to work with fMRI signals and our design protocols. Multivariate GCM was applied to multiple time-series of selected ROIs (varying from 5–10) at three different stages of regulation under consideration.

Two important measures of connectivity, namely, *causal density* and *causal flow* were adapted from Seth (2005) and Seth and Edelman (2007) for comparison of functional connectivity across feedback training runs.

**Causal Density**: The *causal density* (*cd*) of a functional network is defined as the fraction of interactions among ROIs that are causally significant. Causal density is given by the relation *cd = gc*/(*n*(*n* − 1)), where *gc* is the total number of causal connections observed and *n* is the network size. A set of unconnected ROIs will have low *cd*.

**Causal Flow**: The *causal flow* (*cf*) of an ROI *i* in Granger-causality graph is defined as difference between the number of outgoing connections and the number of incoming connections. An ROI with highly positive *cf* exerts a strong causal influence over the network and so acts as a *causal source*. An ROI with a negative *cf* can be called a *causal sink* of the network.

## Results

**Figure 2:**
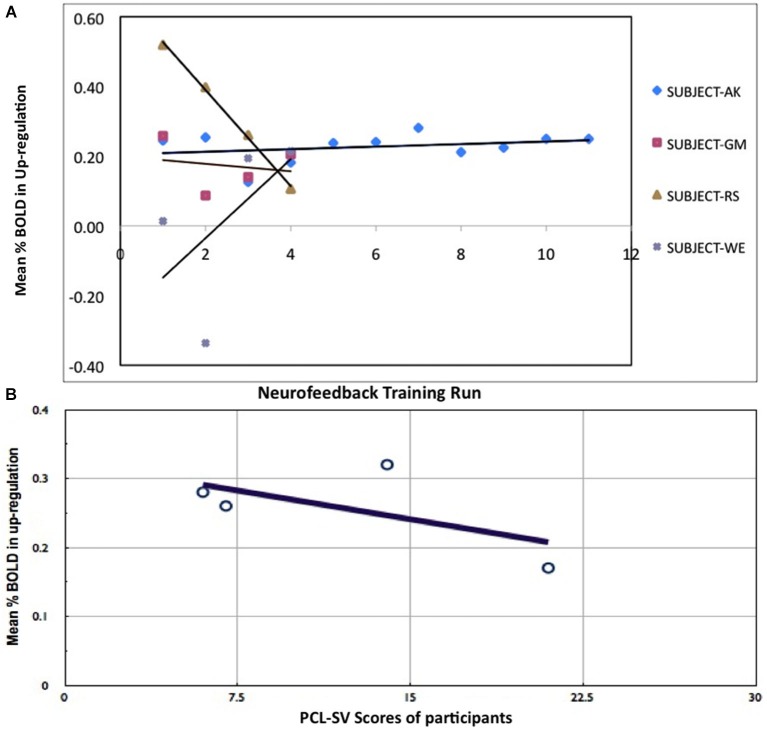
**Results of rtfMRI training. (A)** Percent BOLD change between up-regulation and baseline in all participants across training runs. **(B)** Relationship between psychopathy (PCL:SV) ratings and percent BOLD increase during up-regulation. Negative correlation of percent BOLD change with PCL:SV scores of the subjects (Pearson Correlation = −0.7), indicating that subjects with higher Psychopathic Checklist-Revised (PCL:SV) scores are less successful at self-regulation than their lower PCL:SV counterparts, when compared across equal number of training runs.

Participants reported using the following mental imageries for up-regulation of BOLD signal in the anterior insula: fight with the landlord, death of parents, experience in jail, memories of grand mother, negative memories of a stay in a detention center and pronouncement of judgment in the courtroom. We used the percent BOLD difference between up-regulation and baseline rather than actual values in order to rule out effects of baseline drift and inter-subject differences. Only one participant (Subject-AK) learned to increase the BOLD activity in the anterior insula with training (Figure 2A), while the other participants did not increase their activation levels and in some cases even reduced them (subjects RS and GM). Mean BOLD change in the left anterior insula in the up-regulation condition across the training runs for all participants correlated negatively with the participants’ PCL:SV ratings (Pearson Correlation = −0.7, Figure 2B). Only subject AK had the most consistent increase of BOLD in the up-regulation blocks, and also underwent the most number of training runs (12 runs in 4 days) compared to (4 runs in 1 day) other participants (Figure 2B). To correct for the difference in the training runs, we recalculated the correlation using only data from first four training runs, the corrected Pearson Correlation Coefficient is −0.67.

**Figure 3:**
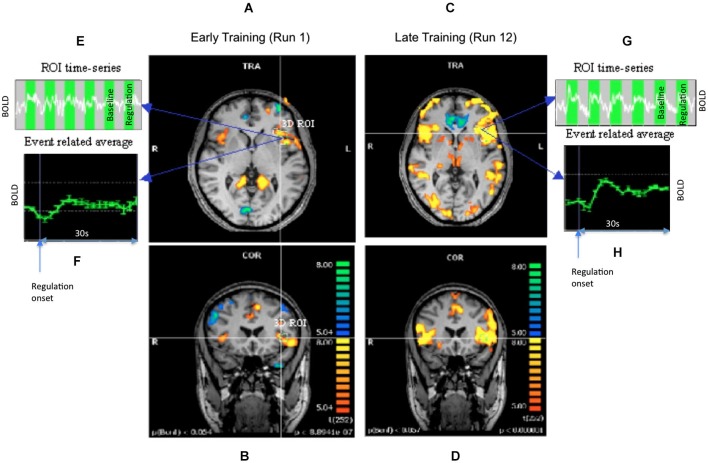
**Results of offline SPM analysis for representative subject AK**. Statistical parametric maps showing the contrast up-regulation vs. baseline during early and late runs. Panels **(A,B)** show the transversal and coronal views of the contrast during an early run when the regulation was still weak. Panels **(C,D)** show the same views for a later run when a stronger regulation was achieved. Maps are all obtained at the same height threshold (*P* = 0.05, Bonferroni corrected). A precise, 3-dimensional ROI was delineated in the left anterior insula. Panels **(E,F)** show the time-series of BOLD signal and its event related average (ERA) in the ROI during weak regulation. The up- and baseline blocks are shown as alternating green and gray rectangles in the time-series. The ERA plots the percent BOLD change in the up-regulation block with respect to the baseline block after averaging across all the blocks of the run. The panels **(G,H)** show similar plots for the stronger regulation.

Because, only subject AK showed consistent activation increases in the up-regulation blocks, further analysis on behavioral data, and effective connectivity from fMRI data were performed in this participant alone. Whole brain analysis for participant AK showed an increased activation cluster in the left anterior insula in the last run when the subject had learned to regulate strongly in comparison to the first day run. The Talairach coordinate for the activated cluster of the left anterior insula for subject AK was (−37, 27, 0; Figure 3).

**Figure 4:**
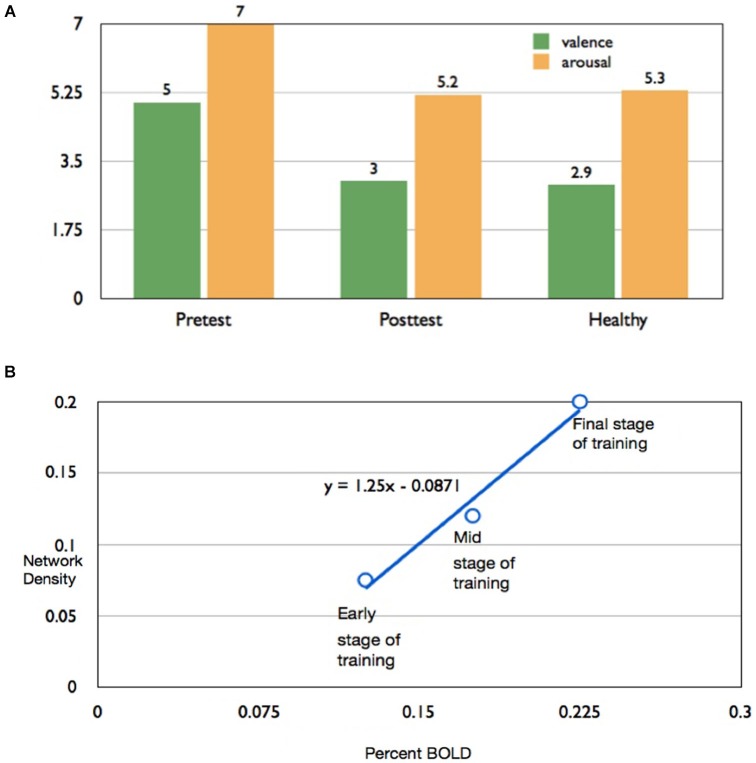
**Effect of rtfMRI training on subjective ratings of aversive pictures** and **(A)** Bar chart of valence and arousal to aversive ratings of subject AK to pictures from the IAPS presented immediately after up-regulation blocks, in the pretest (left bar graphs) and posttest (middle bar graphs), in comparison with mean ratings for healthy individuals reported by Lang et al. (2008). **(B)** Scatter plot showing high correlation (*r* = 0.987) percent BOLD increase in insula and the corresponding values for density of the functional connectivity of the network computed by the Granger Causality Mapping (GCM) method.

For subject AK, mean subjective ratings of valence and arousal of aversive images from the IAPS, after all the up-regulation conditions, decreased from the pretest to the posttest, and became closer in value to the healthy subject ratings reported by Lang et al. (2008) (Figure 4A). This indicates that in the pretest the participant rated aversive images to be more positive in emotional valence and more arousing compared to the standard healthy ratings for the same images. However, during the posttest (conducted after neurofeedback training), the same participant rated the images as being less positive (more negative) and less arousing, and similar to the IAPS healthy ratings.

In the successful subject AK, percent BOLD increase and the *causal density* (*cd*; the number of interactions that are causally significant in the brain network involved in up-regulation) increased proportionately, from the early stage to the final stage of training, in the subject AK (Figure 4B). A high correlation coefficient of 0.987 was observed between the percent BOLD and the causal density.

**Figure 5:**
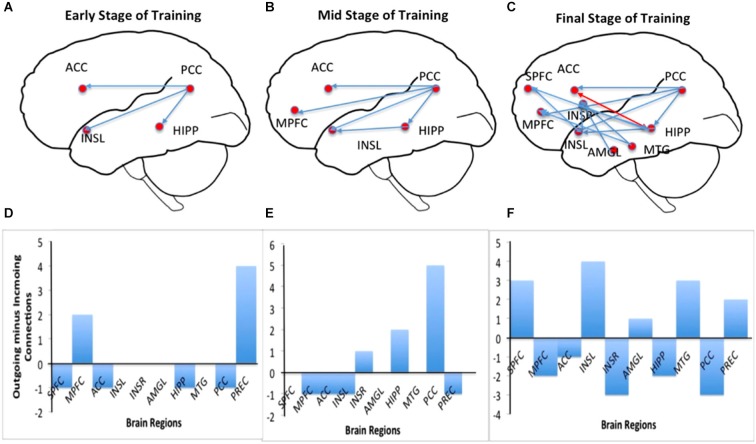
**Effective connectivity analysis of self-regulation of insula for subject AK**. Panels **(A–C)** show directed influence maps (DIMs) and panels **(D–F)** show the bar charts of the causal flow (CF; defined as the net difference between outgoing and incoming connections) in the brain regions (the emotional network) involved in the self-regulation of insula. Panels are arranged from top to bottom from the early through mid to the final stage of training: from early stage of training (top), mid stage of training (middle) to final stage of training (bottom). As the training proceeds, the number of directed influences in the emotional network also increases as shown by the DIMs. During the final stage of training, many regions in the emotional network, including medial prefrontal cortex (MPFC), right insula, anterior cingulate cortex (ACC), amygdala and posterior cingulate cortex are seen to become connected with insula. The CF diagram of the early stage shows that the activations are driven predominantly from the posterior portion of the brain (especially by the posterior cingulate cortex) and that the CF in the left insula is close to zero. However, more training, insula and the anterior regions of the brain seem to drive the network activation to a greater extent as shown by their increasing CF.

Figure 5 shows results of connectivity analysis for three different strengths of regulation in the successful subject AK. Left column of the figure contains schematic depictions of the directed influences among different ROIs; the right column shows bar charts of causal flow (*cf*) for the ROIs.

**Connectivity during the early stage of training**: Figures 5A,D show the brain network and the causal flow, respectively, during the early stage (2nd training run) of the subject AK. Regulation at this stage is driven by the posterior part of the brain, mainly involving the posterior cingulate, which has significant directed influences to left insula, anterior cingulate cortex (ACC) and hippocampus. The posterior cingulate is the major causal source at this stage followed by the left medial prefrontal cortex. At this stage one can see a very sparse network involving interactions among very few ROIs in the emotional network indicated by the low causal density.

**Connectivity during the mid stage of training**: Figures 5B,E show the brain network and the causal flow, respectively, during mid stage of training (5th training run) of the subject AK. Regulation at this stage continues to be driven by the posterior cingulate, which has significant directed influences to left insula, ACC, medial prefrontal cortex and hippocampus. In addition, the hippocampus directs its influence to the left insula. The posterior cingulate is still the major causal source followed by hippocampus at this stage. The causal density of the network has slightly increased compared to the early stage of training.

**Connectivity during the final stage of training**: Figures 5C,F show the brain network and the causal flow, respectively, during the final stage of training (11th training run) of the subject AK. Now, the anterior portion of the brain drives the network, with left insula taking a major share. Left insula is the predominant causal source with directed influences to superior medial frontal cortex, right insula, medial prefrontal cortex and posterior cingulate. Right insula and the posterior cingulate are the major causal sinks. At this stage, a great number of ROIs in the network (high causal density) have been recruited into the regulation function, mainly mediated by the left insula, superior medial frontal cortex and hippocampus. The anterior cingulate now has reciprocal connections with hippocampus. New influences from midtemporal gyrus and amygdale towards insula are also observed.

## Discussion

This pilot study explored the possibility of training criminal psychopaths to volitionally control the BOLD signal in the left anterior insula with the help of an fMRI Brain-Computer Interface developed in our laboratory (Sitaram et al., 2009, 2011). Our previous studies with healthy volunteers (Caria et al., 2007) had shown that learned control of the anterior insula was specific to the region, and not a result of general arousal and global unspecific brain activation, as demonstrated by a control group trained with a non-contingent feedback. From a follow-up study (Caria et al., 2010), we had reported that regulation of left anterior insula modulates the emotional response specific to aversive pictures but not neutral picture stimuli. Both studies had shown that mental imagery alone is not sufficient, and that real-time feedback of the BOLD signal extracted from the target region enhances participants’ ability to achieve regulation. These studies naturally led to the question as to whether patients suffering from clinical conditions characterized by emotion deficits could learn to volitionally regulate anterior insula. In the present study, four criminal psychopaths underwent rtfMRI neurofeedback training. Three psychopathic criminals could only complete four runs of training and were not very successful in learning to control the BOLD signal in the insula. Only one participant (AK) participated in extending neurofeedback training (12 in total) and also learned to successfully control the insula.

In comparison to the low success rate of psychopathic criminals to up-regulate the anterior insula in this study, an earlier study in our lab in 15 healthy participants showed significant increase in the BOLD signal in the anterior insula with five neurofeedback training runs (Caria et al., 2007). Linear regression across all runs showed significant increase of activity in the anterior insula [***y*** = 0.174 + 0.127, ***P*** < 0.012]. Percent BOLD signal change in the region between up-regulation and baseline averaged across all the participants resulted in a clear monotonic increase across the first three runs (repeated measures ANOVA, ***F***_(2,7)_ = 10.32, ***P*** = 0.001). In a subsequent study in our lab, a group of healthy individuals given real-time, veridical feedback learned to increase BOLD activity in bilateral anterior insula while two groups of healthy participants, one with sham feedback and another with no feedback did not learn to increase activity in the region (Caria et al., 2010).

In comparison, the results of the current study, although modest in the number of participants being successful, are still in line with a recent study from our laboratory in schizophrenia patients, and indicates that insula self-regulation can be achieved with fMRI-BCI, even in severe chronic brain disorders (Ruiz et al., 2013b).

A number of reasons could have potentially affected learning in the majority of our psychopathic participants: general lack of motivation to participate in the experiments in spite of the monetary reward, failure to understand and follow experimental instructions, inability to perform emotional imagery and lack of attention to the task. In more basic terms, the inability to learn up-regulation of insula could be attributed to faulty modulation of associative links between external stimuli and internal reactions (Patrick, 1994; Patrick et al., 1994). As per earlier reports, the absence of conditioned fear in psychopathic individuals and the reduced activation of the fear circuitry in the brain (i.e., insula, anterior cingulate, amygdala, orbital frontal cortex, Birbaumer et al., 2005) may be the fundamental reason for the ineffective learning.

In the present study, one psychopathic criminal learned to regulate anterior insula by employing negative emotional imageries in conjunction with contingent feedback, despite a widespread scientific view that the psychopathic brain is resistant to change. We have also extended previous experimental protocols by providing monetary reward after every run. Monetary reward was computed proportional to the percent BOLD increase in the target region to motivate these difficult-to-recruit experimental subjects to continue feedback training. In the successful participant, continued training enhanced the percent differential BOLD in the up-regulation condition compared to the down-regulation condition. Overall, subjects with higher PCL:SV scores were less successful at up-regulation than their lower PCL:SV counterparts. The severity of psychopathic features and different symptom clusters of psychopathy as measured by the PCL:SV have been linked to different emotional processing deficits (Hicks and Patrick, 2006; Hansen et al., 2008; Gao et al., 2012). The current results, although limited due to the results from a single participant, are in line with these studies and with the existing notion that psychopathy consists of a neurobiological trait deficit of aversive anticipatory conditioning responsible for socialization and regulation of negative affect and instrumental aggression (Birbaumer et al., 2005). Alternatively, as psychopathic traits have been associated with insula abnormalities (de Oliveira-Souza et al., 2008; Tiihonen et al., 2008; Schiffer et al., 2011; Ly et al., 2012), the difficulty to learn self-regulation in individuals with higher PCL:SV scores could reflect the abnormality of the insular structure, hypothesis that can be tested in future studies. Although observed in only criminal psychopath, it is nevertheless interesting that the volitional up-regulation of insula was associated with changes in the subjective ratings of valence and arousal of aversive stimuli in this participant. More tests are needed to confirm this finding. This behavioral modulation is congruent with the evidence from previous studies in healthy participants (Caria et al., 2007, 2010) indicating that insula self-regulation modifies the appraisal of emotional picture stimuli, particularly of negative valence. Considering the abnormal autonomic reactivity to aversive stimuli, consistently reported in literature on psychopathy (Lorber, 2004; Gao and Raine, 2010), it would be interesting to assess whether the observed changes in the appraisal of emotional stimuli are accompanied by modifications of physiological measures related to anxiety or other emotional states (e.g., electrodermal response, heart rate, and so forth).

The main purpose of the present study was not only to ascertain whether criminal psychopaths can learn to regulate the BOLD signal in the left anterior insula but also to investigate the changes in effective connectivity in the brain of criminal psychopaths due to successful regulation. In particular, we wanted to compare the effective connectivity changes with varying strength of the regulation. To this end, we employed the multivariate GCM adapted from the Causal Connectivity Toolkit from Seth (2005) to work with fMRI data. A recent study on insula self-regulation showed that fMRI-BCI training is associated with an enhancement in the numbers of effective connections in the emotional network in a successful regulation run, compared with an unsuccessful regulation run (Ruiz et al., 2013b). In the present study, multivariate GCM was applied to time-series of 8–10 ROIs from the emotional network separately for three different levels of regulation, namely, weak, intermediate and strong, determined based on the average percent BOLD increase in up-regulation blocks compared down-regulation blocks. The results show that, firstly, the level of up-regulation is proportional to the causal density of the network, in other words, the extent of “connectedness” of the functional network purportedly involved in the regulation process. Secondly, the results show that during early training, up-regulation is driven mainly from the posterior cingulate, which acts as the main causal source, while right insula and the posterior cingulate are the major causal sinks. With more training, the source of the network moves towards the anterior of the brain finally settling in the left anterior insula during the final stage of up-regulation. During the final stage of training, the left insula directs its influence outwardly to superior medial frontal cortex, right insula, medial prefrontal cortex and posterior cingulate, indicated by the high value of the causal flow. In addition, the hippocampus, amygdala and midtemporal gyrus are introduced into the causal network during the final stage of training. Our results are supported by previous reports of emotional regulation and processing. Ochsner et al. (2004) have shown that up- and down-regulating negative emotion recruited prefrontal and anterior cingulate regions implicated in cognitive control. Further, they reported that self-focussed regulation recruited medial prefrontal regions implicated in internally focused processing, whereas situation-focussed regulation recruited lateral prefrontal regions implicated in externally focused processing. In our study, the predominance of medial prefrontal activation and its involvement in the causal network, and in addition the self-report of regulation strategies by subjects (e.g., fight with the landlord, death of parents, experience in jail, memories of grand mother, negative memories of a stay in a detention center and pronouncement of judgment in the courtroom) indicate the employment of self-focussed imagery, and thus further corroborate Ochsner’s findings of the critical role of medial prefrontal cortex in self-focussed emotional appraisal (Ochsner et al., 2004).

The modulation of brain functional connectivity and the enhanced involvement of frontal areas during the final stage of training could have important implications. In fact, several recent studies have reported abnormal neural functional connectivity in psychopathy. Particularly, a reduced functional connectivity in prefrontal-amygdala circuits have been repeatedly observed (Marsh et al., 2011; Motzkin et al., 2011; Osumi et al., 2012; Juárez et al., 2013; Contreras-Rodríguez et al., 2014). The present findings suggest that fMRI-BCI could serve to modulate abnormally activated cortical and subcortical brain circuits, including brain systems purportedly abnormal in psychopaths, e.g., “the circuitry of empathy” (Decety and Svetlova, 2012; Decety et al., 2012). Interestingly, Ly et al. (2012) reported that psychopath inmates exhibit a reduction in functional connectivity between insula and the dorsal ACC, which could be a neural correlate of the abnormalities in the flexibility of goal-directed attention observed in psychopath. In light of this, future studies for training control of functional or effective connectivity between brain regions, e.g., insula and ACC, could serve to explore specific hypothesis regarding the link between neural substrates and psychopathology (Ruiz et al., 2013a).

Our results question a position emphasizing the resistance to change of psychopathic traits and its heritability. If the brain defect in psychopathy (evident from the neuroimaging literature on classical aversive conditioning) is genetically determined, then the modification of that circuit would not be possible at all. Our pilot study indicates to the contrary that criminal psychopaths could learn to volitionally control brain activity pertaining to emotion and that might have brain and behavioral consequences. Of particular interest are the results of GCM in the course of regulation improvement. The more a psychopathic individual regains control over the deficient activity of the anterior insular cortex, the greater is the restoration of the brain network of emotion.

The study has limitations. Firstly, there are obvious challenges to recruiting this very special population of study participants, and then motivating them to undergo multiple days of neurofeedback training. Secondly, it is difficult to ascertain how much of the variability observed in the capability to self-regulate can be explained by differences in motivation, compliance with the instructions, and other factors that might be playing a role in a population with psychopathic traits. Also, as a preliminary report, no long-term behavioral effects were explored. Future studies with larger samples will serve to generalize our results.

In summary, this pilot study shows modest feasibility that criminal psychopaths can learn self-regulation of a circumscribed brain area with fMRI-BCI, leading to modifications in the functional emotional network. Considering the limited evidence of effective treatment of psychopathy (Salekin et al., 2010) this proof-of principle study suggests that fMRI-BCI could offer a new valuable tool for modulating abnormal brain activations and behavior in this severe condition.

## Conflict of interest statement

The authors declare that the research was conducted in the absence of any commercial or financial relationships that could be construed as a potential conflict of interest.
